# Effects of foreign language learning on executive functions in healthy older adults: study protocol for a randomised controlled trial

**DOI:** 10.1186/s12877-021-02051-x

**Published:** 2021-02-15

**Authors:** Judith Alina Grossmann, Verena Magdalena Koelsch, Merve Gul Degirmenci, Steffen Aschenbrenner, Birgit Teichmann, Patric Meyer

**Affiliations:** 1 Network Aging Research, Heidelberg University, Heidelberg, Germany; 2 Department of Clinical Psychology and Neuropsychology, SRH Clinic Karlsbad-Langensteinbach, Karlsbad, Germany; 3 Heidelberg University Hospital, Heidelberg, Germany; 4 SRH University Heidelberg, Heidelberg, Germany

**Keywords:** Foreign language training, Cognitive reserve, Cognition, Attentional control, Inhibition, Updating, Shifting, Verbal fluency

## Abstract

**Background:**

With age, most cognitive functions decline. As the number of people aged 60 years and older is expected to rise rapidly within the next decades, identifying interventions that promote healthy cognitive ageing is of utmost importance. Promising research on bilingualism has led to the notion that learning a foreign language could protect against cognitive decline. Foreign language learning likely promotes executive functions, which are higher-order cognitive abilities particularly affected by age-related cognitive decline. However, evidence is still sparse and has produced contradictory results. This study aims to investigate the effects of short and intensive foreign language learning on executive functions in healthy older adults.

**Methods:**

In a randomised controlled trial, we will assign 60 native German-speaking monolingual healthy older adults, aged 65–80 years, to either a foreign language learning or a waiting list control group. Language learners will attend a face-to-face, group-based Spanish course for beginners for 1.5 h a day, 5 days a week, for a total of 3 weeks. Cognitive performance in executive functions will be assessed before and after the intervention or after a waiting period of 3 weeks (waiting list control group). Participants will be tested again after 3 months to evaluate longitudinal effects of foreign language learning. The waiting list control group will receive Spanish lessons only after the final assessment and will be invited to an additional voluntary evaluation after completion of the course.

**Discussion:**

To the best of our knowledge, we are conducting the first randomised controlled trial on the effects of short and intensive foreign language learning in older adulthood on executive functions. Enhanced cognitive performance after foreign language learning would indicate that learning a foreign language could enlarge cognitive reserve and thus promote healthy cognitive ageing in older adults.

**Trial registration:**

German Clinical Trials Register DRKS00016552. Registered on 11 February 2019.

## Background

The number of people aged 60 years or older will more than double worldwide to around two billion by 2050 [1]. With age, the majority of cognitive functions decline [2]. This not only threatens individual independence but can also enter pathological stages such as mild cognitive impairment and dementia, in its most severe form [3]. Cognitive ageing concerns a wide range of cognitive functions. Among these, executive functions (EF) are most markedly affected [2]. EF are a set of higher-order cognitive abilities that are involved in the control of mental activities and behaviour and are predominantly seated in the prefrontal cortex [4]. However, cognitive decline proceeds not linearly, because it is impacted by numerous variables. These include not only genetic and medical [5], but also psychosocial factors such as education and intelligence [6]. Moreover, cognitive benefits have already been documented for a variety of cognitively stimulating mental activities in older age such as playing the piano [7] or video gaming [8] (see [9] for a review, see also [10]). Mental activity builds up a cognitive reserve, which is a presumed mechanism for coping with age-related brain damage through a more effective and flexible use of cognitive networks [11]. Interest in foreign language learning as a cognitively stimulating mental activity later in life has only recently emerged and could constitute a promising approach to benefit cognitive functioning.

The idea of investigating foreign language learning as a powerful candidate for enhancing cognition in later life originated from research on bilingualism. Bilingualism can be defined as the ability to speak two languages fluently [12]. It has been associated with better cognitive performance compared to monolinguals, particularly in EF [13, 14]. Moreover, bilinguals exhibit slower cognitive ageing [15]. This so-called ‘bilingual advantage’ in EF probably arises from the demands placed on the cognitive control system by handling two languages. For example, although only one language is being used, both are always simultaneously active in the brain. Therefore, interference caused by the irrelevant language must be inhibited (see [16] for a review). Evidently, foreign language learning differs from bilingualism in important aspects, e.g. in the degree of proficiency. Nevertheless, foreign language learning is likely to promote EF in a comparable way, since language learning also engages an extensive cortical and sub-cortical brain network [17, 18]. This network is responsible for the control of both linguistic and non-linguistic information [19], and overlaps with the network affected by cognitive decline [20]. In initial stages of foreign language acquisition, the left inferior frontal gyrus of the prefrontal cortex is likely to play a major role [18]. Especially when it comes to producing the weaker (foreign) language, this gyrus is involved in the regulation of automatic processes that require attentional control. These processes include inhibition, interference or response selection and suppression [17]. Importantly, attentional dysregulation is probably also contributing to age-related deficits in other cognitive domains such as task switching or response competition [2]. For this reason, foreign language learning could enhance cognitive functions that are particularly affected by cognitive decline.

So far, however, research on cognitive outcomes of foreign language learning in older age is still sparse and has produced mixed results. In a small review summarising results of studies involving a foreign language training intervention [21], five papers [22–26] investigated cognitive outcomes before and after a language course intervention. After adding three more recently published studies to this overview [27–29], no clear cognitive advantage of foreign language learning became evident. Half of the studies detected benefits in one or more cognitive domains, including attentional switching [26], inhibition [24], working memory [29], and global cognition [27, 29]. In contrast, others found no effect on spatial and verbal intelligence [22], working memory [22], task switching [23], and global cognition [25, 28]. This inconsistent picture could be due to several reasons. Overall, there was little overlap between cognitive domains and tasks applied among studies. Interestingly, studies focusing on EF tasks that required high amounts of attentional control were among those showing significant improvements in cognition after foreign language learning [24, 26]. Studies also differed considerably in their effectiveness depending on the design of the intervention. For example, studies with shorter (e.g. 1 week [26]) but higher intensity interventions (at least 5 h of training per week, spread over several days a week) [24, 26] were more consistently associated with cognitive improvements than studies with longer but less intensive interventions [25, 28]. Thus, studies with a higher-intensity foreign language training might be more likely to affect cognition. It is also important to note that some previous studies exhibited limitations, e.g. lack of a control group [24, 25], no randomised group allocation [23, 26], lack of follow-up periods [22–25, 27, 28] and missing control of cognitive impairment [24–26]. These should be considered in future studies. We therefore decided to conduct a randomised controlled trial with high-intensity training and comprehensive control of cognitive impairment to determine whether foreign language learning can improve EF in older adults.

## Methods/design

### Aims

The objective of the present study is to assess the effects of short and intensive foreign language learning on EF in healthy older adults. We hypothesise that, compared to a passive control group, foreign language learning improves EF (hypothesis 1), and that these effects persist in the long-term over 3 months after the intervention (hypothesis 2). To test our hypotheses, we will focus primarily on two tasks affording a high degree of attentional control, since foreign language learning is likely to address this fundamental process of EF. However, as this field still is largely unexplored, we will additionally consider a wider range of EF, including inhibition, shifting, and updating, as the most commonly accepted EF [30]. Moreover, we will evaluate domains that can broadly be classified as EF, but which equally cover linguistic functions (verbal fluency) and non-executive components of attention.

To further explore our results, we will also assess the role of cognitive reserve, as different levels of cognitive reserve have been shown to influence training-related changes in global cognition [31]. This might also apply for previous foreign language knowledge and usage. Therefore, we assume that both the degree of cognitive reserve (hypothesis 3) and prior foreign language knowledge and usage (hypothesis 4) can predict changes in cognitive scores.

### Study design and setting

The present study is a randomised controlled superiority trial with two parallel groups which investigates the effects of a three-week-long foreign language course on EF in healthy older adults. Sixty community-dwelling older adults will be randomised in a 1:1 ratio to one of two study arms: a language learning group (LLG) and a waiting list control group (WLCG). Data will be collected at the Network Aging Research (NAR), Heidelberg University, Germany and the SRH University Heidelberg, Germany. This study protocol follows the Consolidated Standard of Reporting Trials (CONSORT) statement [32] and is presented according to the Standard Protocol Items: Recommendations for Interventional Trials (SPIRIT) guidelines [33].

### Recruitment

Participants will be recruited from the general public in Heidelberg, Germany, and the surrounding regions through advertisements in local newspapers, advertising in trains, at public lectures, and on the NAR homepage. We will also inform individuals who agreed to be notified about ongoing studies via an e-mailing list of the NAR. Additionally, we will distribute flyers and posters in senior and sports centres, language schools, medical practices, and pharmacies.

### Eligibility criteria

Eligibility criteria for participation are listed in Table 1.

**Table 1:** Eligibility criteria for participation

Inclusion criteria	Aged between 65 and 80 years
	Native language German
	Living independently
	Motivated to participate; Signing written informed consent for study participation confirmed by the local ethics committee
Exclusion criteria	Spanish level ≥ A1.1 according to the Joint European Reference Frame for Languages or having already attended a regular Spanish course for beginners (≥ 18 h of teaching)
	Bilingualism or multilingualism (def.: fluent command [level ≥ C1 as defined by questions derived from the Joint European Reference Frame for Languages] and frequent usage of any foreign language)
	Any Romanic language (French, Italian, Latin, Portuguese) level ≥ B1 (as defined by questions derived from the Joint European Reference Frame for Languages)
	Cognitive functions below cut-off (Cognitive Functions Dementia [CFD] [34, 35]: no subtest *z* ≤ − 1.5 [36]) according to age and where possible additionally to sex and education
	Impaired/not-corrected vision
	Colour blindness
	Wearing a hearing aid or impaired sense of hearing as measured by the whispered voice test [37]
	Part-time employment with 20 or more hours of working activity per week
	Self-reported former or current neurological disease (e.g. stroke, mild cognitive impairment, dementia, Parkinson’s disease, epilepsy, multiple sclerosis, encephalitis, cerebral tumour)
	Self-reported current/diagnosed mental health disorder (e.g. anxiety disorder, major depression, schizophrenia, alcoholism or other addiction)
	Transient loss of consciousness for more than five minutes
	Current musical activity for more than five hours per week
	Psychotropic medication within the last six months before the start of the study
	Surgery within the last month before the start of the study
	Other constraints hindering attendance at both the assessments and intervention appointments

### Interventions

This study comprises two trial arms: a LLG and a WLCG.

#### Language learning group (LLG)

The LLG will study Spanish in a three-week course for beginners. Daily lessons of 90 min will be scheduled in the morning from Monday until Friday. In sum, participants will receive 7.5 h of teaching per week, resulting in a total course duration of 22.5 h. The idea of a relatively short but high-intensity training stems from a study by Bak et al. [26], who found a positive effect of foreign language learning on attentional switching after only 1 week of 14 h of foreign language instruction.

A qualified teacher will give the lessons face-to-face at a language school in the centre of Heidelberg, which is easily accessible by car and public transportation. Beyond attending regular classes, participants may do homework and will be allowed to practice at home to consolidate the newly learned content. Following the example of previous studies [23, 38], we will limit the group size to a maximum of ten participants per group, to enable the teacher to address all participants and to give them sufficient opportunity to participate in class. A commonly used work book in adult education [39] will serve as teaching material. By the end of the course, approximately three chapters will have been completed. Participants will learn accent and pronunciation as well as grammatical rules of the Spanish language and will acquire elementary communication skills in various topics (e.g. introducing themselves, asking for directions).

#### Waiting list control group (WLCG)

The WLCG will not receive any treatment during the intervention phase but will take part in a three-week waiting period during which subjects will follow their usual daily routine. To reduce barriers to participation due to possible group preferences [40], the WLCG will attend a control group programme after their study completion. The programme consists of the same language course intervention as the LLG and an additional voluntary examination after the end of the course. This appointment is not included in the main study design as the scope for participation should differ as little as possible between groups.

### Outcomes

**Fig. 1:**
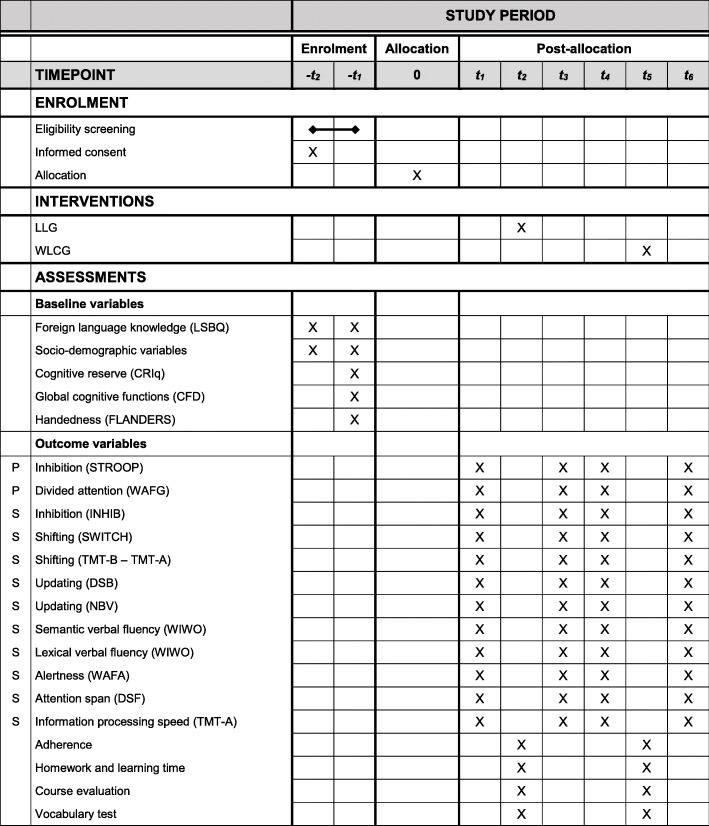
SPIRIT figure for the schedule of enrolment, interventions, and assessments. P primary outcome measure, S secondary outcome measure, −t2 telephone screening, −t1 face-to-face screening, t1 pre-assessment, t2 language course/waiting period, t3 post-assessment, t4 3-month follow-up assessment, t5 language course, t6 4-month follow-up assessment (waiting list control group only). Abbreviations: CFD Cognitive Functions Dementia, CRIq Cognitive Reserve Index questionnaire, DSB Digit Span Backwards, DSF Digit Span Forwards, FLANDERS Flinders Handedness Survey, INHIB Response Inhibition, LLG Language learning group, LSBQ The Language and Social Background Questionnaire, NBV N-Back Verbal, STROOP Stroop Interference Test, SWITCH Task Switching, TMT-A/−B Trail-Making Test – Langensteinbach Version part A/part B, WAFA Perception and Attention Function Battery – Alertness, WAFG Perception and Attention Function Battery – Divided Attention, WIWO Vienna Verbal Fluency Test, WLCG Waiting list control group

Baseline variables, as well as primary, secondary, and language course outcome measures are presented in Fig. 1. Unless otherwise stated, primary and secondary outcome measures are valid and reliable tests from the Vienna Test System (VTS) [34]. The VTS is a computerised test system for the assessment of neuropsychological functions and has become a well-established tool in clinical practice. Each cognitive outcome will begin with an instruction phase, followed by a practice phase, in which participants will receive immediate feedback on their errors. If necessary, the practice phase can be repeated to ensure a sufficient understanding of the task.

#### Baseline variables

Baseline variables include socio-demographic variables such as date of birth, gender, marital and occupational status, as well as an assessment of global cognitive functions. Additionally, cognitive reserve, foreign language knowledge and usage, and handedness will be examined.

##### Cognitive reserve

We will evaluate cognitive reserve using the Cognitive Reserve Index questionnaire (CRIq) [41]. The CRIq is a valid and reliable half-structured interview, which quantifies cognitive reserve over an individual’s lifetime using three established measures of cognitive reserve: education (CRI-Education), occupation (CRI-WorkingActivity), and engagement in cognitively stimulating activities (CRI-LeisureTime) [42]. The three sub-scores can be combined into an overall score (CRI-Index). All measures are age-adjusted to allow for comparisons between different age groups.

##### Foreign language knowledge and usage

Foreign language knowledge and usage will be determined using the Language and Social Background Questionnaire (LSBQ) [43]. The LSBQ is a validated and reliable questionnaire for self-assessment of foreign language skills and usage in various contexts. A composite factor score quantifies the degree of bilingualism on a continuum from clearly monolingual to highly bilingual.

##### Global cognitive functions

We will assess global cognitive functioning using the Cognitive Functions Dementia test set (CFD, test form: S1 [touchscreen operation]) [34, 35] to exclude participants with suspected cognitive decline according to Petersen’s criteria (no subtest *z* ≤ − 1.5) [36].

The CFD is a comprehensive computer-based assessment from the VTS and takes about 60 min to perform. Previous studies in this field of research only used brief screening measures such as the Mini-Mental State Examination (MMSE) [44] or the Montreal Cognitive Assessment (MoCA) [45]. Both instruments can distinguish pathological cognitive decline from healthy ageing. However, they are less accurate than comprehensive multidimensional neuropsychological inventories [46]. In the CFD, cognitive performance is measured by eleven tasks falling into five cognitive domains: attention, verbal long-term memory, EF, expressive speech, and perceptual motor functions.

##### Handedness

The Flinders Handedness survey (FLANDERS) [47] is a short standardised questionnaire to measure hand preference. It contains ten items describing ten different activities (e.g. writing). Participants are asked to indicate whether they prefer the left, the right or either hand for each activity.

#### Primary outcomes

The Stroop Interference Test (STROOP, test form: S7) [48] and the Divided Attention, a subtest of the Perception and Attention Function Battery (WAFG, test form: S3) [49], are the two primary outcome measures.

##### Stroop Interference Test (STROOP)

**Fig. 2:**
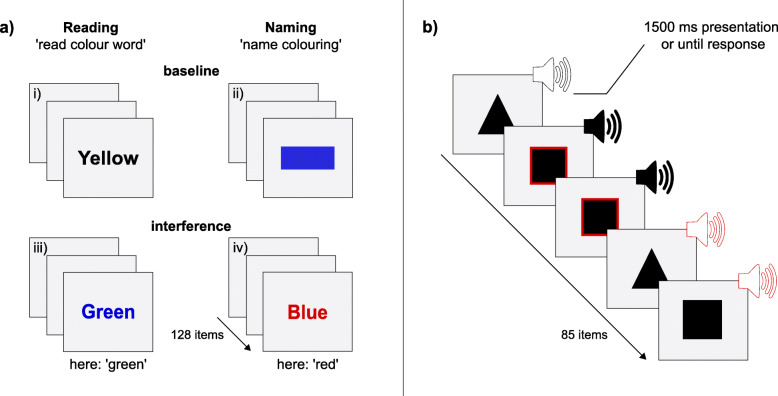
Illustration of primary outcomes, **a)** the Stroop Interference Test (STROOP) and **b)** the Divided Attention (WAFG). White speakers represent the high-pitched sound. Black speakers depict the low-pitched sound. Squares or speakers outlined in red indicate the targets of the visual and the auditory channels. These targets are either two squares or two high-pitched sounds which immediately follow each other

The STROOP is a sensory-motor speed test that determines interference — a measure of inhibitory control. In a small pilot study older adults performed significantly better in this task after having participated in a foreign language training [24]. The task consists of two baseline and two interference conditions (see Fig. 2a). In the (i) reading baseline condition, participants read one of four German colour words (blue, green, red, yellow) printed in black one after the other on a computer screen. Similarly, in the (ii) naming baseline condition, subjects see coloured bars (blue, green, red, yellow). The task is to select the corresponding colour on the response panel as quickly as possible. In the two interference conditions, participants see German colour words printed in a different colour (e.g. the written word ‘yellow’ printed in red). In the (iii) reading interference condition, participants must read the word and ignore the colouring. Conversely, in the (iv) naming interference condition, participants must respond to the colouring and ignore the meaning of the word. Reactions in the two interference conditions are usually slower since the processing of the two simultaneously presented stimuli (the written word and the colour in which it is printed) requires attention. This leads to delayed processing and thus to longer reaction times. Each test part consists of 128 stimuli, which are presented until a response is given. Immediately afterwards, the next stimulus appears. The total test duration is approximately 15 min. The ‘naming interference tendency’ is the primary outcome variable. It is calculated from the difference between ‘median of reaction times – naming interference condition’ and ‘median of reaction times – naming baseline’. Using the median reaction time ensures that asymmetries caused by circumstances, such as ‘getting stuck’ on certain items, do not distort results. The reliability of the naming interference tendency measure is α = .97.

##### Divided Attention (WAFG)

The WAFG is a measure of cross-modal (visual/auditory) divided attention. In the WAFG, subjects are simultaneously confronted with a visual and an auditory channel (see Fig. 2b). On the former one, either a triangle or a square appears on a computer screen. On the auditory one, either a low- or a high-pitched tone is emitted. The task is to press a key on the response panel as quickly as possible when either two squares or two high-pitched tones immediately follow each other. The total number of items is 85, of which 21 are relevant. Stimuli are presented until a response is given or after a maximum of 1500 ms followed by an inter-stimulus interval of 1000 ms. If no reaction has occurred within the presentation period, an omission error is counted. Reaction times below 100 ms will not be considered, as physiological responses below 100 ms are not possible [50]. The total test duration is 9 min. The outcome variable is the logarithmic mean reaction time, which accounts for the expected skewness in the distribution of reaction times. The WAFG, test form S3, obtains a reliability score of α = .84.

#### Secondary outcomes

Secondary outcomes are cognitive measures of EF other than the STROOP and the WAFG. In particular, we will apply tasks from the three core domains of EF: inhibition, shifting, and updating [30]. For comparison reasons, we will also assess verbal fluency and non-executive components of attention. As these predominantly refer to linguistic and attentional functions, they are less likely to load on EF [51, 52]. Details of the respective tasks for each domain of EF and the corresponding dependent variables are presented in Table 2. Every domain is measured by at least two tasks to better capture each construct [60].

**Table 2:** Overview of secondary outcomes

Domain	Task	Test form	Response mode	Description	Dependent variable
Inhibition	INHIB [53]	S3, (go/no-go)	Response panel	A series of circles and triangles is displayed in succession on the screen. Whenever a triangle appears, the subject must press a green button. Circles do not require a response and occur rarely, whereas triangles appear frequently. This builds a dominant response tendency on the triangles. Hence, reactions to circles require inhibition. A total of 250 stimuli are presented, comprising 202 triangles and 48 circles.	Number of commission errors (number of false reactions to circles)
	STROOP [48]	S7	Response panel	The task is described in detail in the primary outcomes section.	Reading interference tendency
Shifting	SWITCH [54]	S1	Response panel	A sequence of triangles and circles appears one by one on the screen. The figures are either dark or light grey. The task is to categorise each stimulus alternately by shape or brightness. After every two stimuli the feature to be attended changes. In case of an incorrect response, the feature to be considered in the next stimulus is displayed (e.g. shape). This hint helps the respondent return to the task after a possible loss of overview. In total, 160 stimuli are presented.	Task switching speed, task switching accuracy
	TMT-B – TMT-A [55]	S2, S4	Touch-screen	This task consists of two parts: A (S2) and B (S4). In part A, numbers from 1 to 25 are randomly displayed on the screen. The task is to connect the numbers in ascending order as quickly as possible. In part B, numbers (1–13) and letters (A – L) are presented. The task is now to alternately link numbers and letters in ascending order (e.g. 1 – A – 2 – B etc.).	Difference score (working time B – A)
Updating	DSB [56]	N/A	Verbal	The test leader is reading a series of numbers aloud. The participants must repeat the numbers in reverse order. The task consists of eight items with a constant increase of one number per item. Each item may be attempted twice. If not at least one sequence of numbers of an item can be repeated correctly, the task stops.	Number of correct trials
	NBV [57]	S1, S3	Response panel	Consonants are displayed one after the other on the screen. If a consonant is identical to the one that has been displayed two places (S1) or three places back (S3), the respondent must react by pressing the green button. S1 contains 100 consonants. In S3, 140 consonants are shown.	Number correct
Verbal fluency	WIWO [58]	S2, S4	Verbal	In these two tasks, the subject must name within two minutes as many words as possible that belong to the category of first names (S2) or begin with the letter K (S4).	Number of correct words
Attention	WAFA [59]	S1	Response panel	Twenty-five black circles are presented one by one on the screen. The task requires to react as quickly as possible by pressing the green button when a circle appears.	Logarithmic mean reaction time
	DSF [56]	N/A	Verbal	The procedure is identical to that of the DSB *(see above)*. The task is to repeat the numbers in the same order.	Number of correct trials
	TMT-A [55]	S2	Touch-screen	*See above.*	Working time

Abbreviations: *DSB* Digit Span Backwards, *DSF* Digit Span Forwards, *INHIB* Response Inhibition; *N/A* Not applicable, *NBV* N-Back Verbal, *STROOP* Stroop Interference Test, *SWITCH* Task Switching, *TMT-A/−B* Trail-Making Test – Langensteinbach Version part A/part B, *WAFA* Perception and Attention Function Battery – Alertness, *WIWO* Vienna Verbal Fluency Test

##### Inhibition

Inhibition is the ability to suppress unwanted reactions. It will be assessed using the Response Inhibition (INHIB) [53] and the ‘reading interference tendency’, which is another outcome measure of the STROOP, as already described in the Primary outcomes section.

##### Shifting

Shifting refers to the ability to switch flexibly between tasks or mental sets. We will measure shifting using the SWITCH [54] and the Trail-Making Test – Langensteinbach Version part B minus part A (TMT-B – TMT-A) [55].

##### Updating

Updating (as a core working memory process) reflects the ability to maintain and continuously update information. It will be evaluated using the Digit Span Backwards (DSB) from the Wechsler Adult Intelligence Scale – Fourth Edition (WAIS-IV) [56] and the N-Back Verbal (NBV) [57].

##### Verbal fluency

Verbal fluency is defined as a person’s ability to find words of a specific characteristic in the mental lexicon [51]. To assess verbal fluency, we will conduct the Vienna Verbal Fluency Test (WIWO) [58]. The test distinguishes between two dimensions: semantic and lexical verbal fluency.

##### Attention

Attention will be represented by a measure of alertness, a basal process of short-term attention activation. It will be measured using the Alertness, a subtest of the Perception and Attention Function Battery (WAFA) [59]. Attention span and information processing speed will be assessed using the Digit Span Forwards (DSF) from the WAIS-IV [56] and the TMT-A [55], respectively.

#### Language course outcomes

We will collect the following language course outcomes to evaluate the intervention:

##### Adherence

Adherence to the language course will be documented daily by the Spanish language teacher. The teacher will inform the study team if a participant does not show up for class without giving prior notice.

##### Homework and learning time

We will collect information about the time spent on additional learning activities in Spanish at home. In a table, participants will indicate in minutes, how much time they invested each day, including at weekends, in doing homework and voluntary learning activities in Spanish. Records will start from the beginning of the course and will end on the last day of the course. The outcome is the total time spent on additional learning activities at home.

##### Course evaluation

A questionnaire will be completed anonymously on the last day of the course to evaluate the perceived quality and acceptability of the intervention. On a four-point Likert scale from ‘fully agree’ to ‘fully disagree’ or ‘cannot judge’, participants will give their opinions on the lessons, the teacher, the textbook, their degree of motivation, and their general satisfaction with the course. Additionally, three open format questions allow participants to provide more specific feedback on aspects they appreciated, disliked, or would recommend for improvement.

##### Vocabulary test

To assess the degree of acquired Spanish language skills, participants will take a vocabulary test on the last day of the course. Following the example of Berggren et al. [22], the vocabulary test will include 108 out of around 577 words learnt during class. Two points will be awarded if the word translated from German into Spanish is translated and spelt correctly. One point will be granted if it has the correct meaning but is misspelt, ignoring punctuation. If the translation is wrong or no answer is given, zero points will be assigned. Thus, a maximum of 216 points can be achieved.

### Participant timeline

**Fig. 3:**
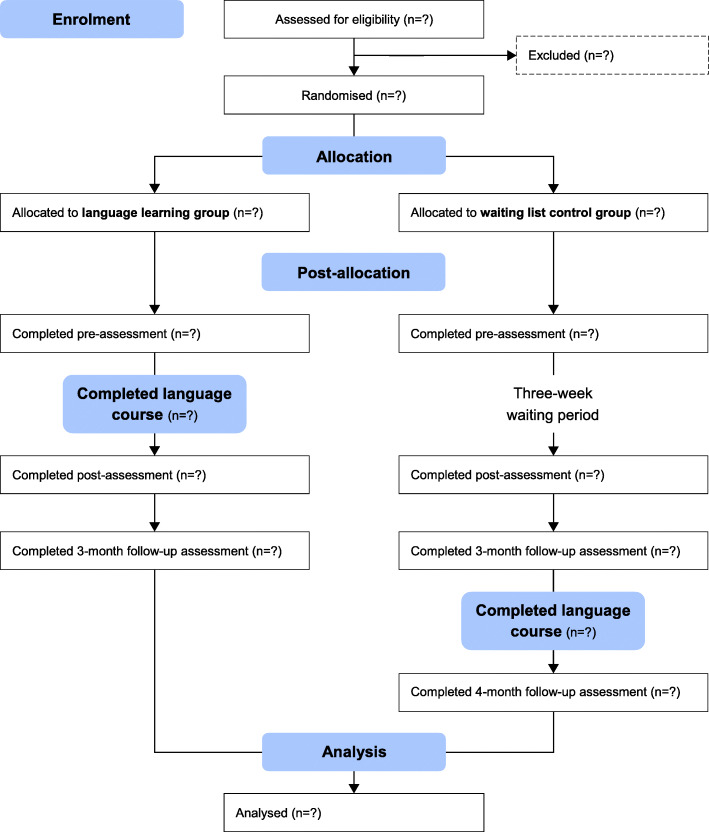
CONSORT flow diagram of participants

Figure 3 illustrates the flow of participants through the trial. Eligibility for participation will be ascertained via a telephone and a subsequent face-to-face screening. In the telephone screening, initial inclusion and exclusion criteria regarding demographic, medical and health-related aspects (see Table 1) as well as availability to participate in the intervention and assessments will be clarified. Moreover, self-reported foreign language skills and usage will be surveyed. Participants who claim to possess few previous skills in Spanish and have not yet attained level A1.1, or who have attended less than one regular course (< 18 h of classes), will be asked a list of questions to determine their Spanish skills. The questions are derived from units 1–5 of the course book [39]. An appointment for the face-to-face screening will be scheduled if they meet all eligibility criteria clarified by telephone.

The face-to-face screening will take around 2 h and will evaluate the remaining exclusion criteria. The CFD will be applied to exclude participants with suspected cognitive decline and the whispered voice test [37] will be used to ascertain sufficient hearing. Participants who indicated in the telephone screening to already have slight experiences in Spanish will take a placement test [61] to ensure that their previous skills are low enough (< level A1.1) for a Spanish course for beginners. Additionally, further baseline data will be collected (see section ‘Baseline variables’).

Subjects that meet eligibility criteria after the face-to-face screening will be randomly assigned to either the LLG or the WLCG. Within 2 weeks before the start of the language course or the three-week waiting period, both groups will participate in a pre-assessment. Both post- and 3-month follow-up assessments will be carried out within 2 weeks immediately (post) and 3 months (3-month follow-up) after the end of the intervention or the waiting period. After this, the LLG will have completed study participation while the WLCG will attend the control group programme consisting of the language course and a voluntary 4-month follow-up assessment.

Pre-, post-, and follow-up assessments will last around 2 h each and will be scheduled at the same time of day, either in the morning or in the afternoon, to control for day-dependent variability in cognitive performance. Assessments will include both the primary and secondary outcome measures listed in Fig. 1. The test order will be randomised by a computer for each individual. However, the WAFA, as the most straightforward task, will always be applied first to allow participants to become familiar with the computerised test system.

To enhance participant retention, we will arrange appointments for pre-, post-, and follow-up assessments with each participant individually before the start of the study. A confirmation document with the scheduled dates and times will be sent to participants. Additionally, participants will receive reminder phone calls 1 week before each assessment. There will be no financial compensation apart from the language course, which will be offered for free. However, participants may obtain information about their results and the conclusion of the study after having completed participation.

### Sample size

Sample size calculation was based on the main (first) research question for the two primary outcomes, the STROOP and the WAFG, and was conducted using G*Power (version 3.1.9.2). The alpha level was set to α = .05 with the power to detect significant differences of 0.8. A total of *N* = 42 participants will be needed to detect a medium effect size of *d* = 0.25 [24, 26, 62] between pre- and post-assessment, assuming a correlation between repeated measures of *r* = .5. We will adjust for multiple comparisons using the Bonferroni correction. A total sample size of *N* = 60, with *n* = 30 individuals per group, will be required to account for a 30% drop-out rate [24].

### Allocation and blinding

Participants who provide written informed consent and pass all screening procedures will be randomly assigned at a 1:1 ratio to one of the two trial arms. A researcher (MGD), who is not involved in the implementation of the current trial, will generate the randomisation sequence using a web-based randomisation system [63]. To avoid predictability of group allocation, the system will build permuted blocks of random sizes, namely two, four, six, and eight, with a list length of 20 [33]. The randomisation sequence and the block sizes will not be disclosed to trial implementers. To ensure concealment, MGD will prepare and store sequentially numbered, sealed, opaque envelopes containing information on group allocation for each participant. She will not open the envelope assigned to a participant until he or she has been included in the trial by JAG. JAG will also inform participants by telephone about their group assignment.

Due to the nature of our study design, participants cannot be blinded to treatment allocation. There can also be no blinding of assessors and teachers. However, assessments will be predominantly computer-based and standardised to reduce test leader effects.

### Data collection and management

JAG and trained research assistants will conduct the assessments. To limit the impact of the test leader on results, we aim to ensure that for each participant at least pre- and post-assessments will be conducted by the same assessor. For every assessor, the number of assessments will be balanced between groups.

Data will be saved and stored according to the DSGVO. At the start of the study, every participant will be allotted a unique identification number. Only persons involved in study conduct will have access to the connection code and the data. As assessments will be mainly conducted via computer, most data will be generated and saved automatically. Data from paper documents will be continuously entered into the database throughout the study period and will be kept in a locked cabin. All electronic data will be stored on a secure server and password-protected computers of the NAR. Additional password protection for electronic documents with identifying participant information will be provided. To promote data quality, we will check data randomly throughout study conduct. Missing data will be addressed as quickly as possible. Before the start of the analysis, we will recheck all data for completeness and correctness. We will fully anonymise data upon completion of data collection, at the latest by 31 March 2021.

### Monitoring

Adverse events will be assessed as part of routine monitoring. No formal data monitoring committee will be established, as this study will involve mostly standardised cognitive assessments. Also, foreign language training poses only a minimal risk of adverse events and will be of short duration. The principal author (JAG) is responsible for trial conduct. She will continuously discuss with the immediate trial team all aspects concerning participant safety, study design, and data management. Participants will be informed that they can contact the study team at any time if they have questions or concerns about the study. Any adverse event related or potentially related to study participation will be instantaneously reported to the immediate trial team, the sponsor, and the local ethics committee.

### Statistical analysis

Statistical analysis will be conducted using IBM SPSS Statistics 26 (IBM Corporation: Armonk, NY, USA). We will use the intention-to-treat dataset, considering all participants as randomised who have completed at least pre-assessment and regardless of adherence to protocol. Hypotheses will be tested two-sided on an alpha level of α ≤ .05, which will be adjusted for multiplicity for primary outcomes by applying the Bonferroni correction. Multiple imputation techniques will be applied to account for missing data assuming that data are missing at random (MAR). Additionally, we will report reasons for withdrawal and analyse them qualitatively.

Primary and secondary outcomes will be analysed using 2 (group) × 3 (time) repeated measures analysis of variance (ANOVAs) with group as between-subjects factor and time-point of assessment (pre-, post-, 3-month follow-up) as within-subjects factor. Bonferroni’s post hoc test will be conducted to test hypotheses. We predict a significant group x time interaction, in which the LLG will outperform the WLCG at post- compared to pre-assessment (hypothesis 1) and at 3-month follow-up compared to pre-assessment (hypothesis 2). Partial eta square (*η*_*p*_^2^) will be used as an indicator of effect size.

For the LLG, exploratory subgroup analyses will be performed to evaluate whether effects of foreign language learning on cognitive outcomes are dependent on different levels of cognitive reserve (hypothesis 3) and foreign language knowledge and usage (hypothesis 4). Therefore, we will conduct multiple and simple regression analyses, respectively. The difference in primary outcome measures between pre- and post-assessment will be defined as the dependent variable. Age and indicators of cognitive reserve (CRI-Education, CRI-WorkingActivity, CRI-LeisureTime, and CRI-Index) (hypothesis 3) and the LSBQ-score (hypothesis 4) will be considered as independent variables.

Additionally, we will investigate whether short-term effects of foreign language learning on cognitive measures also apply to the WLCG after completing the control group programme. For this purpose, the WLCG will serve as its control. Using *t*-tests for dependent means, we predict a significant difference between the difference scores from 3- to 4-month follow-up compared to the difference scores from pre- to post-assessment.

To assess the robustness of results, we will perform sensitivity analyses for the following:

- We will examine the influence of protocol non-adherence by comparing the intention-to-treat to the per-protocol dataset. Participants will be included in the per-protocol dataset if they have completed at least pre- and post-assessment and a minimum of 14 h of formal language instruction (> 62% of the total course duration) referring to Bak et al. [26].
- Effects of missing values will be assessed by comparing results from the complete case (only including subjects without any missing values) to the imputed dataset.

### Dissemination

Results will be disseminated regardless of the magnitude or direction of effects. They will be communicated to the scientific community through journal publications and congress presentations. We will also inform participants and interested individuals, groups, and institutions about trial results.

## Discussion

In this randomised controlled trial, we will investigate whether foreign language learning can improve EF in healthy older adults. The very few studies that have addressed this question to date yielded highly contradictory results. Consequently, well-controlled randomised trials are urgently needed in this field. In our study, we will compare changes in EF in healthy older adults after having attended an intensive three-week, face-to-face, group-based Spanish course for beginners (LLG) with a passive control group (WLCG). As primary outcomes, we will focus on two tasks requiring effortful attentional control, the STROOP, a measure of interference, and the WAFG, an assessment of divided attention. Our secondary outcomes will include tasks from the most widely accepted domains of EF – inhibition, shifting, and updating [30]. To extend the spectrum of EF covered and also to highlight the specificity of our results, we will include two additional domains that largely relate to linguistic [51] and attentional functions [52]. We will also investigate the longevity of any cognitive improvement after foreign language learning. Additionally, we will explore whether different levels of cognitive reserve and previous foreign language skills and usage influence our results.

Our study will have several strengths and limitations. One limitation is that the comparator is only a passive control condition. It is included to monitor improvements in cognitive performance through repeated measurements. The use of a passive control as the only comparison group has already been subject of extensive debate. Some authors argue that clinical trials should ideally include an active control of similar duration and intensity to control for effects attributable to specific treatment modalities, e.g. social activity [21, 64]. However, the inclusion of an active comparator depends on the research question. To date, foreign language learning has not provided clear evidence of maintaining or improving cognitive performance in older adults [21]. Thus, from an economic perspective, it is necessary to examine its effectiveness against a passive control before controlling for other influences or demonstrating its superiority over other activities [65]. Therefore, we decided not to include an active comparator. To still be able to differentiate effects of the intervention from more general effects, we included some secondary outcomes which should not benefit from foreign language learning. This can be presumed because verbal fluency and non-executive components of attention largely relate to cognitive functions which might be less affected by foreign language learning. Furthermore, a significant regression of previous foreign language skills and usage on our results could also be informative. Different levels of cognitive reserve have already been associated with changes in cognition after cognitive training [31]. Thus, assuming previous foreign language skills and usage are part of cognitive reserve, varying results depending on prior skills could indicate a direct effect of language training.

To our knowledge, we are conducting the first randomised controlled trial of its kind with a short and intensive foreign language training. We opted for this approach, as more intensive and short foreign language learning programmes were more likely to be associated with cognitive improvements than less intense trainings with longer durations [24–26, 28]. Furthermore, this short intervention period increases our chances of achieving the targeted sample size, since older adults, although retired, often lead an active lifestyle, and thus have limited time resources.

Our work might also contribute to the current debate on the bilingual advantage hypothesis. This hypothesis suggests that bilinguals perform better in cognitive domains involved in language control, particularly in EF, and suffer less cognitive decline compared to monolinguals [66]. However, accumulating evidence challenges this advantage. For example, a recent large-scale meta-analysis of 152 studies could not find any support for a bilingual advantage across all domains of EF after controlling for publication bias [67]. Although intensive foreign language training and bilingualism differ in important aspects (e.g. short-term learning vs. long-term use), we still might learn more about cognitive processes that underlie foreign language learning. This is of considerable importance as the bilingual advantage might only occur under certain circumstances, which are not yet fully understood [68]. Nevertheless, we would like to emphasise that this study was not intended to address the debate on the bilingual advantage. Our study concerns a well-defined pattern of foreign language usage and a low level of language skills. Thus, no general conclusion can be drawn about the bilingual advantage hypothesis, and any comparison with bilingualism should be taken with caution.

Preserving and enhancing cognitive health in older age is highly relevant, given our ageing world population [1]. As Bak [12] has already noted, if foreign language learning turns out to enhance EF in older adults, it might have clear-cut advantages over drugs or other provisions against cognitive decline. Foreign language learning is inexpensive, already widely available and easily combinable with other activities such as music or travelling. Therefore, foreign language learning could constitute an attractive and enjoyable opportunity to promote cognitive reserve, and thus healthy cognitive ageing.

### Trial status

This study is still ongoing. Recruitment began on 1 March 2019 and is expected to continue until 1 July 2020. By the time of submission of this manuscript (31 March 2020), *n* = 54 participants were already enrolled in the trial.

## Data Availability

This document is the full protocol. No public access to the anonymised dataset and the statistical code will be provided. However, both will be available from the corresponding author upon legitimate request.

## Abbreviations

ANOVA: Analysis of variance
CFD: Cognitive Functions Dementia
CONSORT: Consolidated Standard of Reporting Trials
CRIq: Cognitive Reserve Index questionnaire
DSB: Digit Span Backwards
DSF: Digit Span Forwards
EF: Executive functions
FLANDERS: Flinders Handedness Survey
INHIB: Response Inhibition
LLG: Language learning group
LSBQ: The Language and Social Background Questionnaire
MAR: Missing at random
MMSE: Mini-Mental State Examination
MoCA: Montreal Cognitive Assessment
NAR: Network Aging Research
NBV: N-Back Verbal
SPIRIT: Standard Protocol Items: Recommendations for Interventional Trials
STROOP: Stroop Interference Test
SWITCH: Task Switching
TMT-A: Trail-Making Test – Langensteinbach Version Part A
TMT-B: Trail-Making Test – Langensteinbach Version Part B
VTS: Vienna Test System
WAFA: Perception and Attention Function Battery – Alertness
WAFG: Perception and Attention Function Battery – Divided Attention
WAIS-IV: Wechsler Adult Intelligence Scale – Fourth Edition
WIWO: Vienna Verbal Fluency Test
WLCG: Waiting list control group
